# Long-term survival following multidisciplinary treatment of metastatic sarcomatoid renal cell carcinoma: a case report

**DOI:** 10.1186/s13256-015-0751-0

**Published:** 2015-11-19

**Authors:** Hiroshi Yaegashi, Kouji Izumi, Hiroyuki Konaka, Atsushi Mizokami, Mikio Namiki

**Affiliations:** Department of Integrative Cancer Therapy and Urology, Kanazawa University Hospital, 13-1 Takara-machi, Kanazawa, Ishikawa 920-8641 Japan

**Keywords:** Mammalian target of rapamycin inhibitor, Multidisciplinary treatment, Renal cell carcinoma, Sarcomatoid, Tyrosine kinase inhibitor

## Abstract

**Introduction:**

We report the case of a 62-year-old woman diagnosed with sarcomatoid renal cell carcinoma.

**Case presentation:**

A 62-year-old Asian woman presented with macroscopic hematuria. A histological and immunohistochemical study of a tumor biopsy specimen led to a suspected diagnosis of sarcomatoid renal cell carcinoma. She underwent surgical tumor resection that included her left kidney. A histological and immunohistochemical study of the resected tumor confirmed the diagnosis of sarcomatoid renal cell carcinoma. The pathological stage was pT3bpN2, and multiple lung metastases were detected (pT3bpN2cM1; stage IV). Our patient was classified as “poor risk” according to the Memorial Sloan Kettering Cancer Center risk criteria. Interferon-α was administered as adjuvant therapy, and her lung metastases remained stable. However, a computed tomography scan and bone scintigraphy 2 years later revealed multiple bone metastases. External beam radiotherapy was performed for the bone metastases. Despite continuing interferon-α during radiotherapy, multiple skull and liver metastases appeared. Oral administration of the tyrosine kinase inhibitor axitinib was initiated as a second-line therapy, and our patient achieved a stable state for 11 months. As the liver metastases progressed and meningeal dissemination newly appeared, oral administration of the mammalian target of rapamycin inhibitor everolimus was initiated as a third-line therapy. Our patient remains alive 71 months after diagnosis and has maintained a comparatively good quality of life.

**Conclusion:**

A literature review revealed that metastatic sarcomatoid renal cell carcinoma has very poor prognosis, with a survival of <1 year despite systemic therapy. Our patient in this present case achieved long-term survival, a rare incidence worthy of report.

## Introduction

The sarcomatoid variant of renal cell carcinoma (RCC) features a spindle-cell phenotype and has been reported to have a poor prognosis compared with other histological types of RCC [1–3]. Some reports have noted that the prognosis of patients with sarcomatoid RCC (SRCC) is poor even after tyrosine kinase inhibitor treatment [1]. However, we report our experience with a case of metastatic sarcomatoid RCC (mSRCC) in which the patient achieved long-term survival following multidisciplinary treatment. We also discuss the relevant literature.

## Case presentation

A 62-year-old Asian woman visited a urological office in August 2009 with a complaint of macroscopic hematuria. Following an intensive examination, she was suspected to have a left renal tumor, for which she visited our department in September 2009.

Blood biochemistry data indicated anemia, thrombocytosis, prolonged prothrombin time, hyperkalemia, and elevated levels of alkaline phosphatase, gamma-glutamyl transpeptidase, and lactate dehydrogenase. Her total albumin-corrected serum calcium level was within normal limits. A microscopic urine analysis revealed slight microscopic hematuria and pyuria. Urine cytology was negative for malignancy.

**Fig. 1:**
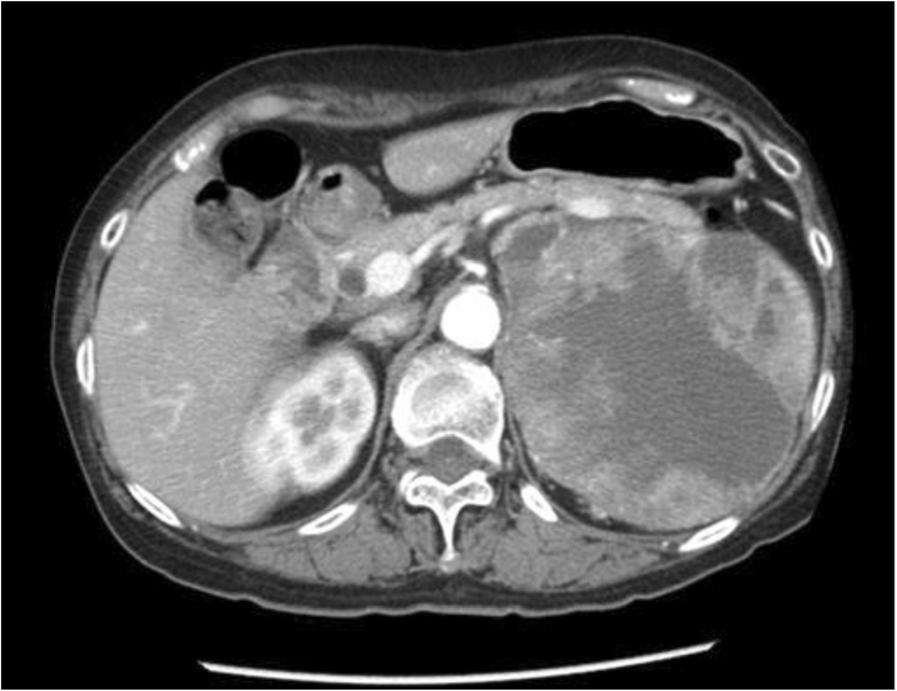
Contrast-enhanced computed tomography examination prior to treatment. A tumor with a 13-cm diameter was detected in the left kidney

Dynamic computed tomography (CT) revealed a tumor with a 13-cm diameter in her left kidney (Fig. 1) and tumor embolism in her left renal vein. Lymph node swelling was also detected at her left renal hilus. In addition, lung metastases were strongly suspected following a lung CT scan.

A CT-guided biopsy of the left renal tumor was performed, and a histopathological examination indicated SRCC. Renography yielded right and left renal function rates of 53.5 mL/min and 30.4 mL/min, respectively. Bone scintigraphy showed no evidence of metastasis. She was diagnosed with a suspected left-side SRCC at clinical stage T3bN2M1 (PUL), according to the TNM classification.

**Fig. 2:**
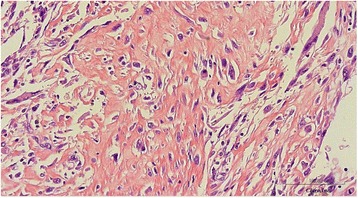
A specimen of the resected tumor. Large, abnormal multinucleated cells or pleomorphic/spindle cancer cells are visible in a hematoxylin-eosin stained tumor section. Magnification ×200

Our patient underwent transabdominal left radical nephrectomy. The pathological diagnosis was SRCC with a >50 % sarcomatoid component; a clear-cell type component was also detected (Fig. 2). The final diagnosis was SRCC (pT3bpN2cM1), and she was considered to be “poor risk” according to the Memorial Sloan Kettering Cancer Center risk criteria.

**Fig. 3:**
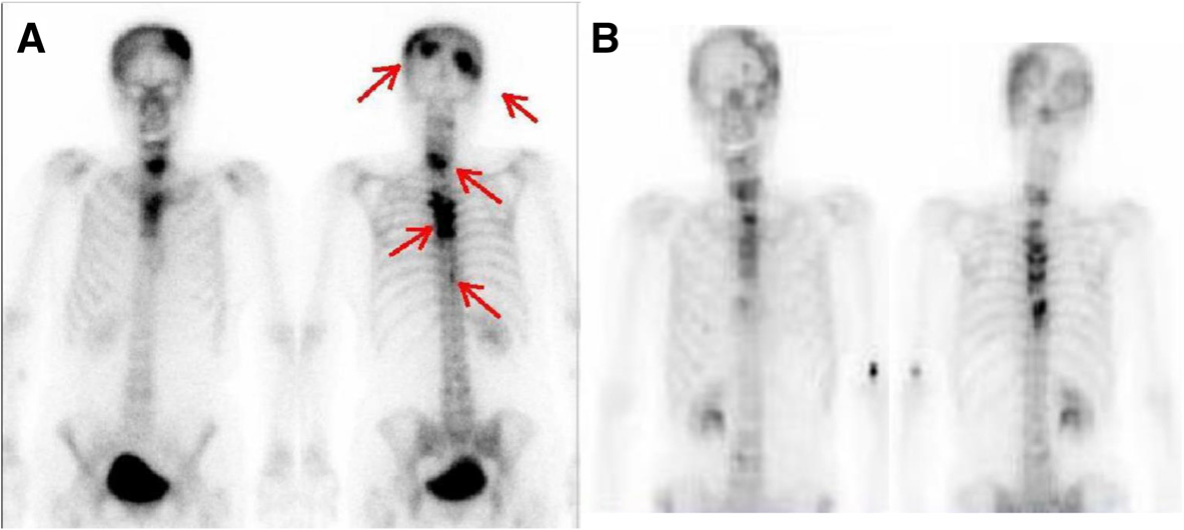
Bone scintigraphy. **a** Bone scintigraphy during interferon-α treatment. Multiple bone metastases are visible (*red arrows*). **b**. Bone scintigraphy at present state. Abnormal accumulations are less visible

Interferon-α (3 million U, three times per week) was initially administrated as an adjuvant systemic therapy. Although the multiple lung metastases subsequently disappeared, a follow-up CT scan 2 years after surgery revealed multiple bone and skull metastases (Fig. 3a). The bone agent zoledronic acid was administrated, and external beam radiotherapy was performed for the bone and skull metastases. Although we were able to control the bone metastases, multiple liver metastases subsequently appeared. We then administered the tyrosine kinase inhibitor axitinib (10 mg/day) as a second-line therapy, as well as denosumab for the bone metastases.

Meningeal dissemination was first detected during follow-up 11 months after switching to axitinib and was treated with stereotactic irradiation. Because our patient’s disease was considered progressive, we administered the mammalian target of rapamycin (mTOR) inhibitor everolimus (10 mg/day) as a third-line therapy. The multiple liver metastases, meningeal dissemination, and multiple bone metastases were well controlled at 16 months after everolimus administration (Fig. 3b). Our patient remains alive with controlled disease 71 months after the initial diagnosis and has maintained a comparatively good quality of life, despite receiving long-term treatment.

## Discussion

SRCC was defined in the 2004 World Health Organization classification of renal tumors as any histologic type of RCC containing foci of high-grade malignant spindle cells. Some evidence indicates an increased risk associated with a sarcomatoid component comprising 5–10 % of total tumor volume [4, 5], suggesting that even a low level of sarcomatoid differentiation might be clinically relevant and should be included in pathology reports.

mSRCC had a very poor prognosis in the pre-targeting therapy era. For example, Molina *et al.* reported 63 cases of sarcomatoid-variant RCC at the Memorial Sloan Kettering Cancer Center, of which 47 were clear cell RCC, and 16 were non-clear cell RCC. The median progression-free and overall survival durations in that study were 3 months and 10 months, respectively, despite the administration of systemic therapy [1].

Zhang *et al.* reported 204 cases of sarcomatoid-variant RCC treated via nephrectomy [2]. Of these, 168 patients died from SRCC at a mean of 1.7 years post-surgery (median 0.8 years; range 0.1–29.4 years). In a further comparison of patients with ≥30 % versus <30 % sarcomatoid differentiation, patients in the former group were significantly more likely to die (hazard ratio 1.52; *p* = 0.018).

In terms of histochemistry, hypoxia-inducible factor (HIF)-1α is well known to induce vascular endothelial growth factor (VEGF), which in turn correlates with tumor progression. Accordingly, Tickoo *et al.* reported high levels of HIF-1α expression in patients with SRCC [4].

On the other hand, some reports have noted the relationship between sarcomatoid features and the epithelial-to-mesenchymal transition (EMT), an important metastatic process, in RCC [3]. For example, Boström *et al.* mentioned that transforming growth factor (TGF)-β expression correlated with EMT in patients with SRCC [6]. Furthermore, Lamouille *et al.* demonstrated a relationship between TGF-β and mTOR complex 2, which is required for cell migration and invasion *in vitro* [7].

Regarding our present case, the clinical course demonstrates the efficacy of switching from interferon-α to molecular-targeting agents. Unfortunately, evidence regarding the effectiveness of axitinib or everolimus for mSRCC is currently lacking.

However, given the fact that VEGF, a target of the multi-tyrosine kinase inhibitor axitinib, is expressed at high levels in SRCC, and that mTOR, a target of everolimus, possibly correlates with EMT in mSRCC, it is reasonable to treat mSRCC with these molecular-targeting agents.

The systemic effects of bone-modifying agents such as zoledronic acid or denosumab on cancer-specific survival remain controversial, and the effects on mSRCC are also unknown. Although the sarcomatoid component comprised >50 % of the RCC in our present case, we were able to maintain a comparatively stable disease through multidisciplinary treatment.

## Conclusions

We have described our experience with a case of mSRCC; to the best of our knowledge, the long survival duration (currently 71 months) is rare. Multidisciplinary treatments, that is, a combination of radical surgery, cytokine therapy, molecular-targeting therapy, radiotherapy, and bone-modifying agent therapy, were therefore effective for long-term survival in this case. Further research is needed to determine the correlation between treatment and molecular mechanisms in mSRCC.

## Consent

Written informed consent was obtained from the patient for publication of this case report and any accompanying images. A copy of the written consent is available for review by the Editor-in-Chief of this journal.

## Abbreviations

CT: computed tomography
EMT: epithelial-to-mesenchymal transition
HIF: hypoxia-inducible factor
mSRCC: metastatic sarcomatoid renal cell carcinoma
mTOR: mammalian target of rapamycin
TGF: transforming growth factor
RCC: renal cell carcinoma
SRCC: sarcomatoid renal cell carcinoma
VEGF: vascular endothelial growth factor

## References

[1] Molina AM, Tickoo SK, Ishill N, Trinos MJ, Schwartz LH, Patil S, et al. Sarcomatoid-variant renal cell carcinoma: treatment outcome and survival in advanced disease. Am J Clin Oncol. 2011;34:454–9. 10.1097/COC.0b013e3181f47aa4 PMC3661202 21127411

[2] Zhang BY, Thompson RH, Lohse CM, Leibovich BC, Boorjian SA, Cheville JC, et al. A novel prognostic model for patients with sarcomatoid renal cell carcinoma. BJU Int. 2015;115:405–11. 10.1111/bju.12781 24730416

[3] Conant JL, Peng Z, Evans MF, Naud S, Cooper K. Sarcomatoid renal cell carcinoma is an example of epithelial--mesenchymal transition. J Clin Pathol. 2011;64:1088–92. 10.1136/jclinpath-2011-200216 22003062

[4] Tickoo SK, Alden D, Olgac S, Fine SW, Russo P, Kondagunta GV, et al. Immunohistochemical expression of hypoxia inducible factor-1alpha and its downstream molecules in sarcomatoid renal cell carcinoma. J Urol. 2007;177:1258–63. 10.1016/j.juro.2006.11.100 17382701

[5] Peralta-Venturina M, Moch H, Amin M, Tamboli P, Hailemariam S, Mihatsch M, et al. Sarcomatoid differentiation in renal cell carcinoma: a study of 101 cases. Am J Surg Pathol. 2001;25:275–84. 10.1097/00000478-200103000-00001 11224597

[6] Boström AK, Möller C, Nilsson E, Elfving P, Axelson H, Johansson ME. Sarcomatoid conversion of clear cell renal cell carcinoma in relation to epithelial-to-mesenchymal transition. Hum Pathol. 2012;43:708–19. 10.1016/j.humpath.2011.06.019 21992819

[7] Lamouille S, Connolly E, Smyth JW, Akhurst RJ, Derynck R. TGF-β-induced activation of mTOR complex 2 drives epithelial-mesenchymal transition and cell invasion. J Cell Sci. 2012;125:1259–73. 10.1242/jcs.095299 PMC3324583 22399812

